# Acoustic-Based Rolling Bearing Fault Diagnosis Using a Co-Prime Circular Microphone Array

**DOI:** 10.3390/s23063050

**Published:** 2023-03-12

**Authors:** Chi Li, Changzheng Chen, Xiaojiao Gu

**Affiliations:** 1 School of Mechanical Engineering, Shenyang University of Technology, Shenyang 110178, China; 2 School of Mechanical Engineering, Shenyang Ligong University, Shenyang 110159, China

**Keywords:** bearing fault diagnosis, acoustic signal analysis, co-prime circular microphone arrays, direction-of-arrival estimation

## Abstract

This study proposes a high-efficiency method using a co-prime circular microphone array (CPCMA) for the bearing fault diagnosis, and discusses the acoustic characteristics of three fault-type signals at different rotation speeds. Due to the close positions of various bearing components, radiation sounds are seriously mixed, and it is challenging to separate the fault features. Direction-of-arrival (DOA) estimation can be used to suppress noise and directionally enhance sound sources of interest; however, classical array configurations usually require a large number of microphones to achieve high accuracy. To address this, a CPCMA is introduced to raise the array’s degrees of freedom in order to reduce the dependence on the microphone numbers and computation complexity. The estimation of signal parameters via rotational invariance techniques (ESPRIT) applied to a CPCMA can quickly figure out the DOA estimation without any prior knowledge. By using the techniques above, a sound source motion-tracking diagnosis method is proposed according to the movement characteristics of impact sound sources for each fault type. Additionally, more precise frequency spectra are obtained, which are used in combination to determine the fault types and locations.

## 1. Introduction

Industrial equipment nowadays has become increasingly large scale and complex. On the other hand, the concept of intelligent manufacturing involves ongoing improvement of equipment performance and resource efficiency. As a result, timely and accurate machine health monitoring plays a far more important role [1]. Bearing failure, which is generally brought on by improper mounting or inappropriate lubrication, is one of the most common causes of a mechanical breakdown, and has consequently led to a remarkable amount of continuous studies [2]. Significant progress has been made in fault diagnosis based on vibration signals employing methods, including time/frequency domain analysis, modal analysis, finite element analysis, etc. [3,4,5]. Techniques such as wavelet transform (WT) and mode-decomposition methods are proposed and effectively extract the information from non-stationary, non-linear signals [6,7].

However, vibration diagnosis still has limitations and cannot work well in cases such as slow rotating and complex dynamics [8]. Acoustic-based diagnosis has gradually developed as a new effective approach. Diagnosis using acoustic emissions (AE) exploits the transient elastic waves when deformation occurs within a material, from which the energy loss could span a wide range of frequencies. AE sensors are able to capture much higher frequencies than vibration sensors, so the technique is more sensitive to early faults [9,10,11,12]. However, AE waves are greatly attenuated during propagation; therefore, just like vibration measurement, AE sensors should be placed as close as possible to the components being tested [13]. Detection based on acoustic radiation signals is another acoustic-based method, which offers the special advantage of non-contact signal acquisition, which is particularly applicable to occasions where sensor attachment is problematic. It also avoids downtime and operational inconveniences induced by sensor installation and maintenance for large equipment [14,15]. Some studies have shown that for certain statistical parameters, acoustic signals may represent clearer features for early defects than vibration signals in rotating machinery fault diagnosis [16,17]. Moreover, acoustic signals are often used in motor fault diagnosis, such as permanent-magnet demagnetization [18], commutator motor with rotor coil or gear defects [19], rotation speed identification, etc. [20]. However, the signal obtained from a single-channel microphone only provides sound pressure values; hence, acoustic measurement is highly sensitive to measuring points and the features of interest are very likely to be obscured by high-level noise, which explains the relative lack of investigation and application in this field [21,22].

Signal processing techniques based on multi-channel microphones have been presented as a solution to the issue. Spatial distribution information of the acoustic field around the sound sources can be reconstructed through the specific positional relationship between microphones, which allows for the directional enhancement and denoising of signals even from the moving sound sources. Near-field acoustic holography (NAH) and beamforming based on far-field signal models are two efficiently used detection techniques. According to Lu [23] and Hou [24], gray-level co-occurrence matrix features were retrieved from sound field images for bearing fault pattern identification. Chen adopted cyclic spectral density as the reconstruction indicator instead of the complex sound pressure to improve the efficiency of the holographic data processing and precisely identified the sources of the stationary sound field [25]. Nejade proposed the multi-reference holography processing method based on residual spectra using a simple structure model and examined its reliability with two industrial machines [26]. Ma developed a vibroacoustic transfer function using the finite element method for multiple rotating components enclosed in a small close space, which was proven to be effective by near-field experiments [27]. Deep learning methods were used in recent works to improve the accuracy of NAH [28,29]. Cabada et al. combined spectral kurtosis (SK) with beamforming to evaluate both the locations and frequencies of bearing failures using a spiral microphone array [30]. Verellen studied the beamformed acoustic data with a variety of signal-to-noise ratios as a reference for ultrasonic fault detection [31]. One of the most typical uses for multi-channel acoustic diagnosis is wayside identification, where researchers have invested large efforts to overcome signal distortion due to the Doppler effect and noise interference [32,33,34,35]. The studies mentioned above basically adopt two research ideas. One is to localize the possible position areas of the defect sound sources, and the other is to filter the signal through directional enhancement. However, microphone arrays provide the characteristic of spatial domain information, which is rarely used in the field of fault diagnosis.

The main purpose of the study is to propose a novel sound source motion-tracking approach for fault-type recognition. Spatial domain information is fully made use of, which provides a fresh perspective on fault diagnosis as an addition to the established analysis methods based on time and frequency domains. Sound sources produced by faults for various components and types are distinct from each other in obedience to the operating mechanism, which gives more logical proofs for diagnosis using motion trajectories indicated by direction-of-arrival (DOA) estimates. DOA estimates are also used for the signal beamforming, and frequency spectra with good noise reduction and obvious fault features can be extracted. The motion trajectories and the spectra work together to identify the fault types, as well as the positions; accordingly, the characteristics of the bearing fault acoustic signals are further observed and analyzed. However, precise DOA estimation of sound sources coming from large equipment often needs to be located in both the vertical and horizontal dimensions. For this reason, microphone configurations such as planar or circular arrays are customarily adopted, and a large number of microphones are used to achieve good accuracy; therefore, the practical applicability of the technique is constrained and subject to an expensive cost and high computational time. The study presents an adaptive array signal processing method that provides high-accuracy direction-of-arrival (DOA) estimation and fault feature extraction with fewer array elements, in order to reduce the reliance on the number of microphones in real applications. For the proposed method, a co-prime circular array (CPCA) configuration is attempted for signal acquisition, and the estimation of signal parameters via the rotational invariance techniques (ESPRIT) algorithm is used for the data processing. The peculiar structure of a co-prime array was first proposed and validated by Xiang and Bush in 2015, which makes it possible to construct virtual array elements. By exploiting the second-order statistics, more degrees of freedom are acquired, which overcomes the Nyquist theorem’s restriction on array apertures [36,37,38,39]. Meanwhile, when using a circular array, ESPRIT exploits the rotational invariance property to obtain paired two-dimensional DOA estimation without any prior knowledge, which avoids the secondary peak-search and accelerates the process speed [40,41,42].

In this paper, acoustic signals from a rolling bearing test rig are collected using a uniform circular microphone array (UCMA), as well as a co-prime circular microphone array (CPCMA) simultaneously, where exactly the same number of microphones are applied in both arrays. Three test conditions are investigated, including outer race fault, inner race fault, and rolling element fault. Signals between the two arrays are observed and compared after the ESPRIT processing, and the effectiveness in fault diagnosis is evaluated.

## 2. Theoretical Background

### 2.1. Co-Prime Circular Array

**Figure 1:**
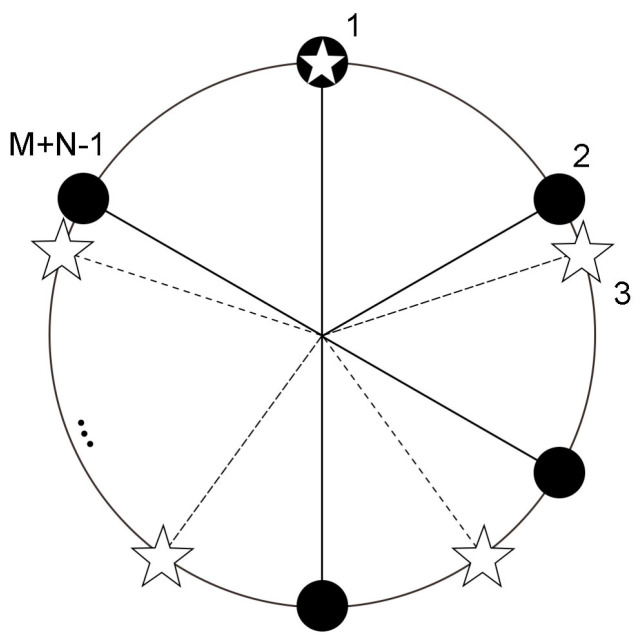
Co−prime circular array (CPCA) configuration.

Co-prime array configuration was at first applied in linear arrays. Let M and N be a pair of co-prime numbers and M < N, then consider two sparse uniform linear sub-arrays with M and N elements, respectively. Overlap the two sub-arrays, and an M + N − 1 element co-prime linear array can be created, which was validated by experiments to have comparable degrees of freedom to a uniform linear array of MN elements [36,37]. The co-prime circular array model is set up based on the co-prime linear array theory. Consider two uniform circular arrays (UCAs) with the same radius as two sub-arrays, in which there are M and N elements, respectively. To form a CPCA, interleave the two sub-arrays. The first element of the new array is shared by the two sub-arrays, as illustrated in Figure 1, where the corresponding array elements of sub-arrays are severally represented by hollow asterisks and filled circles. The number of CPCA array elements is M + N − 1.

**Figure 2:**
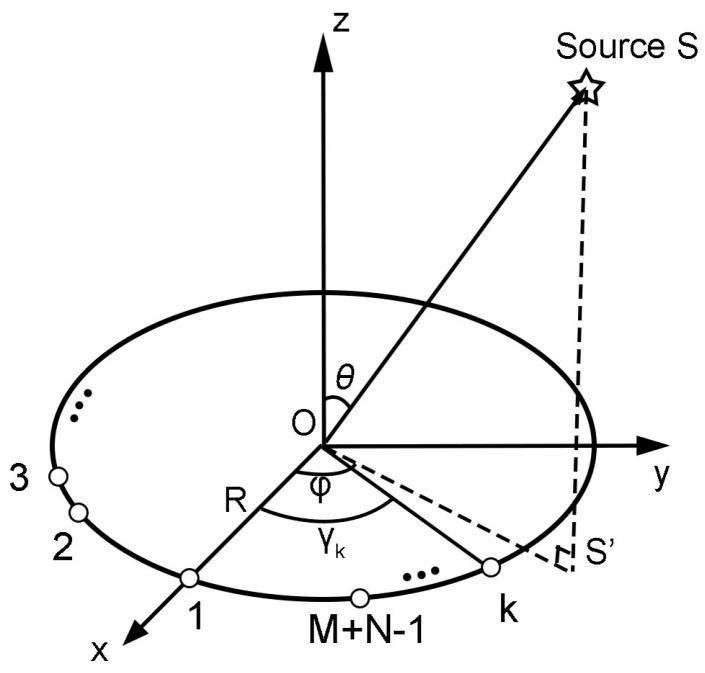
CPCA signal model.

Figure 2 shows the CPCA signal model. Assume a CPCA of radius R in the x−y plane with its elements clockwise distributed over the circumference. Let the origin O of the coordinate system be the center of the array. A narrowband plane wave comes from the sound source S, and S’ is the projection point of S. Take the radius overlaid with the *x*-axis as the reference line passing through Element 1. The angle measured down from the z-axis to OS is the elevation angle θ of the sound arrival direction, and the angle measured from the *x*-axis to OS’ is the azimuth angle ϕ. $\gamma_{k}$ is the angle between the first and the kth element.

The array response vector a(θ,ϕ) can be formulated as:

(1) $$a(\theta ,\phi )=\left[\begin{array}{l} \exp(jkR\sin\theta \cos(\phi -\gamma_{0})\\ \exp(jkR\sin\theta \cos(\phi -\gamma_{1})\\ \ldots \\ \exp(jkR\sin\theta \cos(\phi -\gamma_{M+N-2}) \end{array}\right]$$

When there are P uncorrelated sources, we have:

(2) $$A=\left[a(\theta_{1},\phi_{1}),\ldots ,a(\theta_{P},\phi_{P})\right]$$

The received signal then can be written as:

(3) x(t)=As(t)+n(t)

where s(t) and n(t), respectively, stand for the matrices of the incoming source signal and noise signal.

Then, the covariance matrix of the signal can be obtained as:

(4) $$R_{\mathrm{xx}}=E[x(t)x^{H}(t)]$$

For an M + N−1 element UCA, the angle $\gamma_{k}=2\pi i/(m+n-1),i=0,1,\ldots ,m+n-2$ is easily calculated; however, arc lengths between elements in a CPCA are uneven. To solve this, vectorize $R_{\mathrm{xx}}$ as:

(5) $$vec(R_{\mathrm{xx}})=\left[a^{*}(\theta_{1},\phi_{1})⊗a(\theta_{1},\phi_{1}),\ldots ,a^{*}(\theta_{P},\phi_{P})⊗a(\theta_{P},\phi_{P})\right]p+\sigma_{n}{}^{2}vec(I)$$

where vector p denotes the power squared of the P sources, $\sigma_{n}{}^{2}$ is the noise power, and I is the identity matrix. The vectorization uses difference co-array (DCA) to reconstitute a virtual UCA with MN − 1 uniformly distributed elements, which notably improves the degrees of freedom compared to an M + N − 1 physical element UCA.

### 2.2. UCA-ESPRIT

ESPRIT was initially presented based on uniform linear arrays (ULA). ESPRIT exploits the rotational invariance of the signal subspace; accordingly, the channel manifold vectors should follow the Vandermonde structure, while the UCA signal model does not meet the condition. Transformation based on phase mode excitation converts a UCA manifold vector into a ULA-like Vandermonde matrix by using the recursive nature of Bessel functions. Consider $F_{r}^{H}$ as the beamformer to map the UCA manifold vector a(θ,ϕ) onto the transformed beamspace, whose manifold is defined as b(θ,ϕ). The mapping can be expressed as:

(6) $$b_{}(\theta ,\phi )=F_{r}^{H}a_{}(\theta ,\phi )$$

To keep $F_{r}^{H}$ orthogonal, a rotation angle α is brought in to construct a matrix W that has the structure of centro-Hermitian rows, which is expressed as:

(7) $$W=\frac{1}{\sqrt{K}}\left[v(\alpha_{-K}),\ldots v(\alpha_{0}),\ldots ,v(\alpha_{K})\right]$$

where $\alpha_{i}=2\pi i/K,i\in [-K,K]$. K denotes the highest excited mode passing through the array apertures. v(ϕ) is the vector to convert the azimuth variation into the Vandermonde-like structure, which is given as:

(8) $$v(\phi )={\left[e^{-jK\phi },\ldots ,e^{-j\phi },e^{-j0},e^{j\phi },\ldots ,e^{jK\phi }\right]}^{T}$$

Elevation information of the manifold also needs to be converted into a symmetric amplitude taper, by giving the matrix $C_{V}V^{H}$ that derives from the Bessel function. For a UCA with N elements, we have:

(9) $$C_{V}=diag\left\{j^{-K},\ldots ,j^{-1},j^{0},j^{1},\ldots ,j^{K}\right\}$$

(10) $$V_{}=\sqrt{N}\left[w_{-K},\ldots w_{0},\ldots ,w_{K}\right]$$

where $w_{i},i\in [-K,K]$ is the normalized weight vector for the ith phase mode, which is expressed as:

(11) $$w_{i}=\frac{1}{N}{\left[1,e^{-j2\pi i/N},\ldots ,e^{-j2\pi i(N-1)/N}\right]}^{T}$$

Combined with those aforementioned, the beamformer $F_{r}^{H}$ can be given as:

(12) $$F_{r}^{H}=W^{H}C_{V}V^{H}$$

and the received signal can be converted as:

(13) $$y(t)=F_{r}^{H}x(t)$$

By considering the phase delay between two adjacent array elements as an artificially defined delay matrix, the array can be split into identical sub-arrays. According to the rotational invariance, the sub-arrays share the same signal subspace, which can be obtained by eigenvalue decomposition of the array signal covariance matrix. Combine the sub-array signals into a matrix z(t), the signal subspace S can be calculated by applying eigenvalue decomposition to $R_{\mathrm{zz}}=E[z(t)z^{H}(t)]$ and the eigenvalues of the phase delay matrix Φ can be picked out as well, which gives the paired DOA information θ and ϕ. For P sound sources, there is:

(14) $$\Phi =\mathrm{diag}(\sin\theta_{1}e^{j\phi_{1}},\sin\theta_{2}e^{j\phi_{2}},\ldots ,\sin\theta_{P}e^{j\phi_{P}})$$

## 3. Experimental Investigation

### 3.1. Experimental Setup

**Figure 3:**
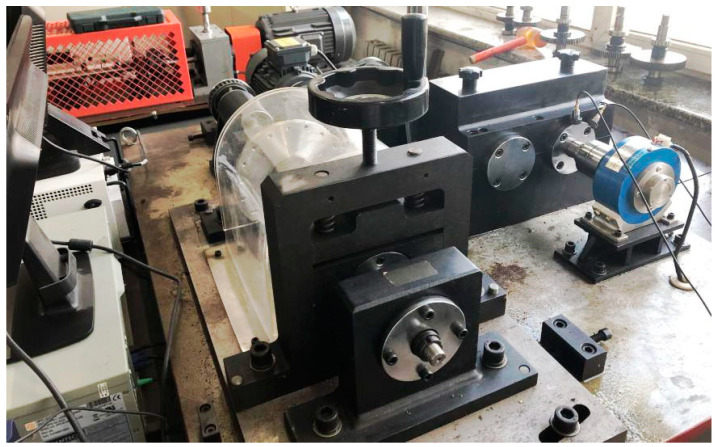
Test rig.

**Figure 4:**
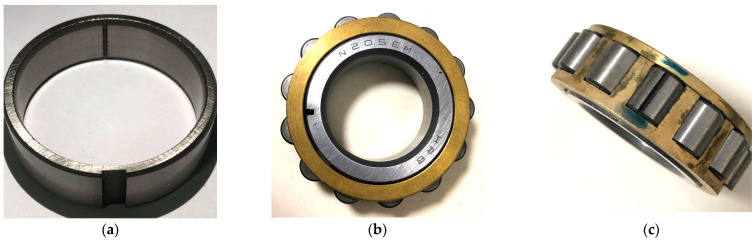
Bearing fault conditions: (**a**) outer race fault; (**b**) inner race fault; (**c**) roller fault.

The experiment is conducted on a QPZZ-II test rig, as shown in Figure 3. The rotor apparatus is driven by an ac motor. The rotation speed is controlled by the generator frequency, which ranges from 75 rpm to 1450 rpm. The bearings (HRB N205EM CHINA) used in the tests are shown in Figure 4. Three test conditions are investigated, including outer race fault, inner race fault, and rolling element fault. When conducting the outer race fault, the outer race breach is mounted at the 5 o’clock position. For each trial, the apparatus operates under constant speeds at 290 rpm and 725 rpm, which corresponds to the generator frequency of 10 Hz and 25 Hz, and the shaft rotational speed of 4.83 Hz and 12.08 Hz. The outer race keeps fixed. Table 1 and Table 2 represent the bearing configuration and the fault feature frequencies.

**Figure 5:**
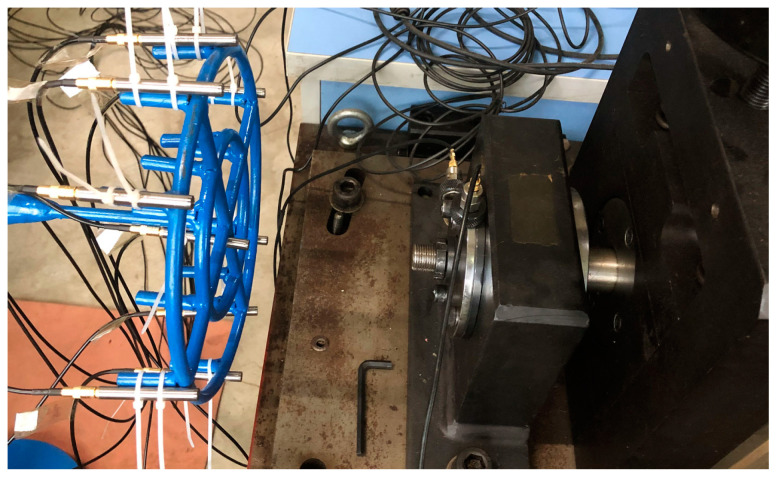
Experimental setup.

**Figure 6:**
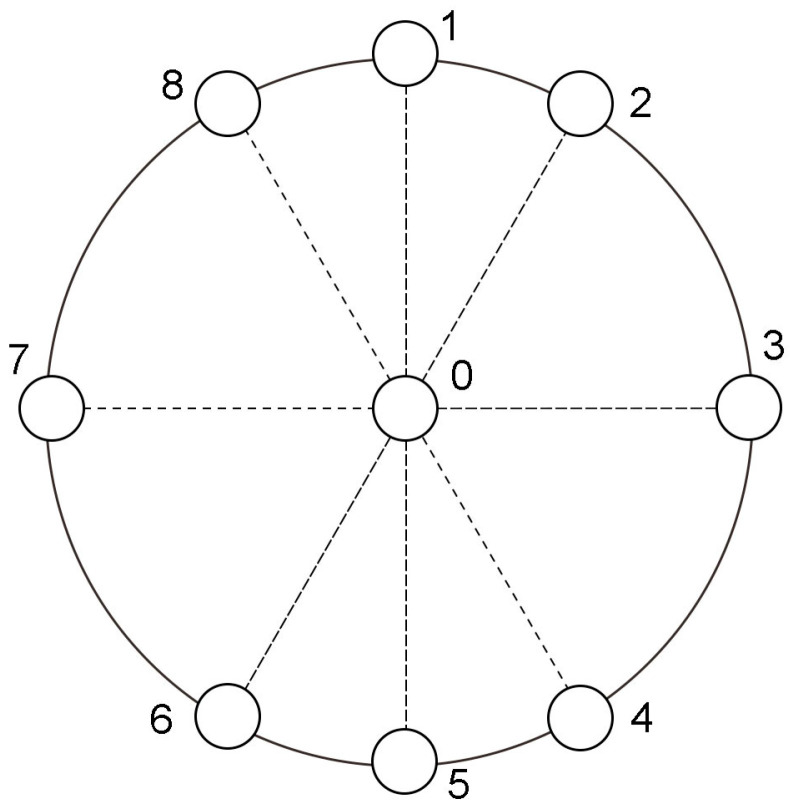
Microphone arrangement.

The microphone array is placed at a 170 mm distance in front of the apparatus, as is shown in Figure 5. The diameter of the circumference is 200 mm. Figure 6 shows the microphone arrangement. A total of nine microphones are used in the measurement. The microphone designated Number 0 is situated at the center to capture mono signals. Numbers 2, 3, 4, 6, 7, and 8 comprise a 6-element UCA, as well as a 3-element co-prime circular sub-array, while Numbers 1, 3, 5, and 7 form another 4-element co-prime circular sub-array, consequently both UCMA and CPCMA have 6 array elements. The setup provides simultaneous signal acquisition of UCMA and CPCMA, which minimizes the effect of environmental variations on the signals collected by both. The data collector is NI PXIe-1082 and the type of microphone is G.R.A.S. 40 ph. For each trial, the signals are recorded at a sample rate of 10.24 kHz during 10 s.

### 3.2. Data Analysis Procedure

Firstly, the motion regularity of each fault type needs to be analyzed. The outer race fault produces sounds when balls are passing through the defect, which are always from the same direction. Inner race fault and rolling element fault generate moving sound sources that uniformly rotate with the shaft. Due to the existence of moving sources, the DOA estimation algorithm cannot be applied directly to a long-lasting data section. The collected data need to be chopped into segments in keeping with the real-time location of the defects.

However, it cannot be guaranteed that every split segment contains information of interest. When there is no collision of defects in the segment, unconcerned sound source positions are likely to be located instead. To address the issue, elevation angles are checked to screen unwanted DOA estimates out, for the impact sounds always come from the circumferences where the defects locate, and the elevation angles from the same circumference have similar values. Azimuth angles then are feasible to be observed to speculate about the fault types. Furthermore, spatial filtering is applied on the basis of the screened DOA estimates, and the resulting frequency spectra also help to determine. The signal processing procedure is carried out as follows:

1. Separate the collected signals into UCMA and CPCMA sets.
2. Figure out the rotation angles between every collision for each defect type. Since the outer race is fixed, the azimuth angle values for the outer race defect should always be the same. To calculate the angular interval of the impact sounds for the inner race defect, we have:

(15) $$\Delta \phi_{ID}=2\pi /(f_{BPFI}/f_{i})$$

where $f_{i}$ is the rotational speed of both the shaft and the inner race.

The rolling element fault has the peculiarity that impact sounds are triggered both on the inner and outer races, and both are moving sound sources, for which we have:

(16) $$\Delta \phi_{RDO}=\pi^{2}d_{B}/(\pi L_{O})$$

(17) $$\Delta \phi_{RDI}=\pi^{2}d_{B}/(\pi d_{I})$$

where $\Delta \phi_{RDO}$ and $\Delta \phi_{RDI}$ denote the rotation angles between defect collisions on the outer and inner races. $d_{B}$ is the nominal diameter of balls, $L_{O}$ is the outer ring land diameter, and $d_{I}$ is the inside diameter of the inner race bore.

An adequate interval angle $\Delta \phi_{L}$ should be determined to divide the data into segments. It is necessary that $\Delta \phi_{L}$ is smaller than $\Delta \phi_{ID}$, $\Delta \phi_{RDO}$, and $\Delta \phi_{RDI}$, so that the positions of impact sounds can be closely tracked. From $\Delta \phi_{L}$, the number of snapshots used to split data can be calculated as:

(18) $$N_{L}=\Delta \phi_{L}L/(2\pi f_{i})$$

where *L* denotes the number of snapshots per second, which depends on the sampling rate of signal acquisition.

**Figure 7:**
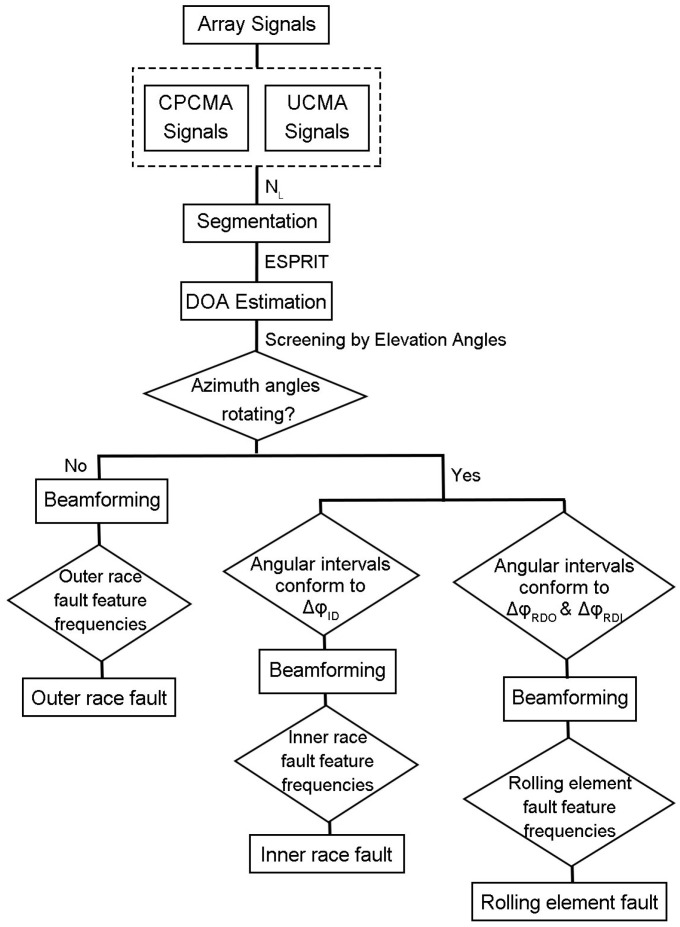
Signal processing and analysis procedure.

3. Apply ESPRIT to UCMA and CPCMA signal segments to obtain DOA estimates of both. Elevation angles for each fault type should be worked out based on the distance between the defect and the reference element of the microphone array. When an elevation angle estimate has a large difference from the calculated value in a segment, it means the segment gives invalid information and the location should be excluded.
4. Compare the azimuth angular intervals of the screened DOA estimates with $\Delta \phi_{ID}$, $\Delta \phi_{RDO}$, and $\Delta \phi_{RDI}$ to observe whether it conforms to the moving regularity of impact sounds for a specific bearing fault type.
5. Apply beamforming for the further determination of fault types.
  The flow chart in Figure 7 represents the data processing and analysis procedure.

### 3.3. Results and Discussion

#### 3.3.1. Outer Race Defect

Table 3 and Table 4 each list a group of screened DOA estimates severally from UCMA and CPCMA signals under the outer race fault condition at 290 rpm. To see clearly, values listed are all expressed in degrees. As mentioned in Section 3.1 and Section 3.2, the breach set on the outer race is placed at a 150-degree angle direction relative to the reference array element, and the elevation angle is calculated as 7.79 degrees. It can be seen that CPCMA has much closer estimates than UCMA. The root-mean-square errors (RMSEs) for each group of values are listed in Table 5.

It is noticed that negative values are occasionally found in the azimuth angle estimates, as has been shown in Table 3. That is because the incident sound waves are presumed to be plane waves in the far-field model, where aliasing in signals may induce a 180-degree phase ambiguity and mislead the estimation results.

**Figure 8:**
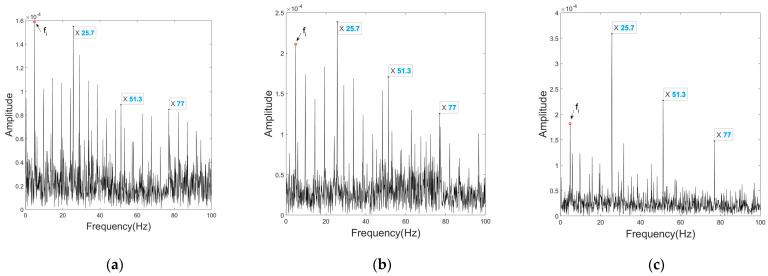
Envelopes under outer race fault condition at 290 rpm of: (**a**) non−processed mono signal; (**b**) UCMA−ESPRIT beamformed signal; (**c**) CPCMA−ESPRIT beamformed signal.

Figure 8 shows the spectral envelopes of the non-processed mono signal, the UCMA-ESPRIT beamformed signal, and the CPCMA-ESPRIT beamformed signal. The fault feature frequencies of the outer race defect are marked.

The mono signal is captured by the microphone at the center of the array right in front of the shaft; therefore fault features are greatly masked by the modulation of the shaft rotational frequency $f_{i}$. The UCMA signal after beamforming presents slightly clearer fault features, but still the modulation of $f_{i}$ causes confusion that cannot be ignored. The beamformed CPCMA signal makes a huge improvement and practically eliminates the interference.

#### 3.3.2. Inner Race Defect

The elevation angle for the inner race defect is calculated as 5.29 degrees. For the inner race fault, Table 6 and Table 7 each list a group of 10 sequential screened DOA estimates at 290 rpm, respectively, from UCMA and CPCMA signals. The trajectory of rotation can be found from the azimuth angle estimates, and in each group, a full rotation is approximately covered. As noted above, values with phase ambiguity likewise come up, which have been marked in the tables.

Table 8 shows RMSE for each group of the estimates. Since the azimuth angle is not a fixed value, the angular interval, which is figured out as 46.42 degrees, is chosen as the observed value in the calculation of RMSE. The results indicate that CPCMA performs better than UCMA at locating, moreover, phase ambiguity appears mainly in UCMA signals, which manifests the same conclusion. However, judging from the elevation angle estimates, both UCMA and CPCMA show a drop in location accuracy when compared with the condition of the outer race fault. It is likely to be induced by the movement of the sound sources.

**Figure 9:**
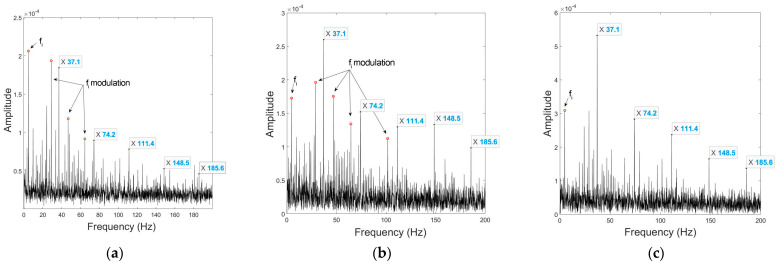
Envelopes under inner race fault condition at 290 rpm of: (**a**) non−processed mono signal; (**b**) UCMA−ESPRIT beamformed signal; (**c**) CPCMA−ESPRIT beamformed signal.

Figure 9 shows a comparison of the spectral envelopes among the three signal processing approaches.

Similar to the outer race fault circumstance, the fault feature eigenfrequency in the mono signal is overwhelmed by the $f_{i}$ modulation, and the harmonics are barely perceptible. Processed UCMA signal roughly extracts fault feature frequencies, yet the interference is still strong and the relative relationship between harmonic amplitudes appears disordered. The issues are all well resolved in the processed CPCMA signal.

#### 3.3.3. Rolling Element Defect

The rolling element defect successively collides with the outer and the inner races, generating fault locating with two different elevation angles. Based on this, DOA estimates after screening are given in Table 9, Table 10, Table 11 and Table 12. Each table lists 10 segments at 290 rpm. Table 13 and Table 14 give RMSEs in the ball pass location of the outer and inner races, where calculated values of the elevation angles remain the same as before, and angular intervals are figured out as 29.03 degrees for the outer race and 54 degrees for the inner race.

It can be found from the DOA estimates of UCMA that elevation angle values are highly dispersed, and the phase ambiguity occurs more frequently, which shows a further decrease in location accuracy compared with the inner race fault condition. It is probably because the interval time between impacts is so close, which aggravates the aliasing of sound waves and makes locating more difficult. The RMSE values confirm the observation that location errors of UCMA are much larger than that under the inner race fault condition. DOA estimation precision of CPCMA also has a slight decline, which is manifested as a small rise in phase ambiguity occurrence frequency. Regardless of this, CPCMA still maintains an accuracy numerically no less than before.

**Figure 10:**
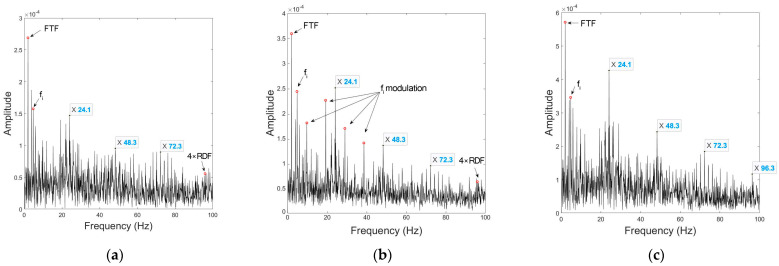
Envelopes under rolling element fault condition at 290 rpm of: (**a**) non−processed mono signal; (**b**) UCMA−ESPRIT beamformed signal; (**c**) CPCMA−ESPRIT beamformed signal.

Figure 10 shows the spectral envelopes of the three signal processing methods, where the rolling element defect frequency (RDF) is equivalent to twice the ball spin frequency (BSF). The spectra of the rolling element fault contain significant fundamental train frequency (FTF) components. Because of the intense interference of its modulation, fault feature frequencies are almost completely submerged and hardly distinguished. UCMA has enhanced the fault features, but due to the inaccuracy of elevation angle estimates, $f_{i}$ is enhanced at the same time, of which the modulation constitutes a new disturbance. In the CPCMA signal, the modulation of both FTF and $f_{i}$ are effectively suppressed as a result of the precise location, and harmonics up to the fourth order are clearly extracted.

#### 3.3.4. Experimental Results at a Different Rotation Speed

**Figure 11:**
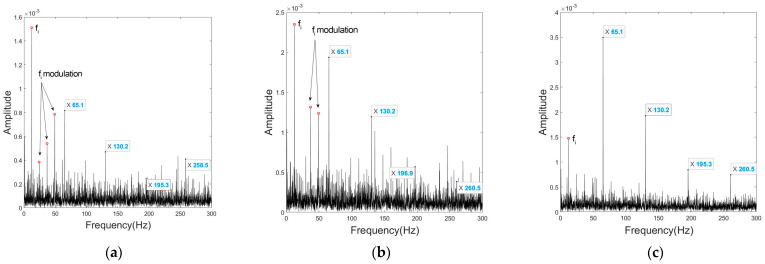
Envelopes under outer race fault condition at 725 rpm of: (**a**) non−processed mono signal; (**b**) UCMA−ESPRIT beamformed signal; (**c**) CPCMA−ESPRIT beamformed signal.

**Figure 12:**
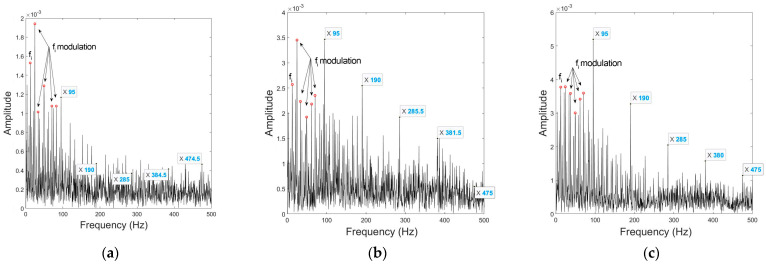
Envelopes under inner race fault condition at 725 rpm of: (**a**) non−processed mono signal; (**b**) UCMA−ESPRIT beamformed signal; (**c**) CPCMA−ESPRIT beamformed signal.

In order to compare the diagnostic efficacy at different rotation speeds, experimental results under the outer and inner race fault conditions at 725 rpm are presented to show the location and fault feature extraction effects of both static and moving impact sound sources. For the outer race fault signal, Table 15, Table 16 and Table 17 give the DOA estimates and RMSE, and Figure 11 shows the spectral envelopes of each processing method. Additionally, Table 18, Table 19 and Table 20 and Figure 12 present the results under the inner race fault condition.

The comparison shows that at a higher speed, the characteristics of both defect and interference sound sources are enhanced, which is mainly evidenced by the amplitude increase in the high-order harmonics of the fault feature frequencies, as well as the $f_{i}$ modulation. From the DOA estimates, elevation angle location accuracies for both fault types are all improved at a higher speed, which is caused by the enhancement of the direct sounds from defects to microphones. However, as can be seen from DOA estimates for the outer race fault, both UCMA and CPCMA show an overall deviation in the azimuth angle localization. This is probably because azimuth location is highly influenced by the reflected sounds, and higher sound pressure of the sources leads to stronger reflections and coherence, which results in location bias. On the other hand, the enhancement of the impact sounds has reduced the dispersion in DOA estimates. It can be presumed that the same is true for the inner race fault, but compared with UCMA, the CPCMA signal maintains almost as good accuracy in angular interval estimates as at 290 rpm.

## 4. Conclusions

In this paper, a novel diagnosis method based on sound source motion tracking is proposed, and acoustic signals of three different bearing fault types at two rotation speeds are acquired to validate its effectiveness. Compared with vibration signals, acoustic signals have significantly more noise and aliasing problems. Due to the close positions of each bearing component, eigenfrequency modulation of the fault-free components, such as the shaft, has a great effect even on the high-order harmonics and in many cases overwhelms the fault feature frequencies. Sounds of the defects can be directionally enhanced by locating the collisions using a microphone array. However, UCMA requires a large number of microphones to achieve a certain location accuracy. CPCMA takes advantage of the non-uniform differential relationship between array elements to create a virtual array through the vectorization process, which considerably raises the degree of freedom of the array and lessens the dependence on the number of microphones needed for high-precision. The superiority of CPCMA performance with the same number of microphones has been verified by experiments in the study. When applying more microphones, the performance gap between the two array configurations will be larger in accordance with CPCA properties. On this basis, using ESPRIT as the signal processing method, the paired elevation and azimuth angles can be quickly estimated, which can largely improve the process speed for array signals. The CPCMA-ESPRIT method realizes the low-cost and high-efficiency sound source location of defects, and satisfies the requirement for intelligent analysis in practical engineering.

## Figures and Tables

**Table 1:** Bearing Configuration.

Pitch Diameter	Ball Diameter	Ball Number	Contact Angle
39 mm	7.5 mm	13	0°

**Table 2:** Bearing Fault Feature Frequencies at 290 rpm.

BPFO ^1^	BPFI ^2^	BSF ^3^	FTF ^4^
25.375 Hz	37.46 Hz	12.102 Hz	1.952 Hz

^1^ Ball pass frequency outer race; ^2^ ball pass frequency inner race; ^3^ ball spin frequency; ^4^ fundamental train frequency.

**Table 3:** DOA estimation by UCMA-ESPRIT for outer race fault signal at 290 rpm.

Elevation Angle (°)	7.08	7.16	7.19	7.04	7.21	8.38	8.10
Azimuth Angle (°)	155.29	145.56	157.75	145.95	−31.17	145.83	−32.81

**Table 4:** DOA estimation by CPCMA-ESPRIT for outer race fault signal at 290 rpm.

Elevation Angle (°)	7.98	7.56	7.70	7.89	7.65	7.95	7.54
Azimuth Angle (°)	145.43	150.45	150.36	151.62	151.95	152.10	149.99

**Table 5:** Root-mean-square error (RMSE) in outer race defect locatied by both arrays at 290 rpm.

UCMA		CPCMA	
Elevation Angle	Azimuth Angle	Elevation Angle	Azimuth Angle
0.6099	4.8820	0.1752	2.1399

**Table 6:** DOA estimation by UCMA-ESPRIT for inner race fault signal at 290 rpm.

Elevation Angle (°)	7.69	6.33	5.89	7.95	6.79
Azimuth Angle (°)	−126.25 ^1^	−81.67 ^1^	−37.17	10.25	59.89
Elevation Angle (°)	4.31	4.73	4.06	4.58	7.24
Azimuth Angle (°)	105.16 ^1^	150.68	−161.60 ^1^	−119.72 ^1^	−78.96 ^1^

^1^ Phase ambiguity exists.

**Table 7:** DOA estimation by CPCMA-ESPRIT for inner race fault signal at 290 rpm.

Elevation Angle (°)	7.02	6.31	5.17	4.90	5.80
Azimuth Angle (°)	−129.24	−82.83	−36.45	8.65	58.31
Elevation Angle (°)	5.85	4.77	5.28	5.29	7.14
Azimuth Angle (°)	106.19	152.59	−162.07	−119.56	−72.64

**Table 8:** RMSE in inner race defect located by both arrays at 290 rpm.

UCMA		CPCMA	
Elevation Angle	Angle Interval	Elevation Angle	Angle Interval
1.5947	2.8828	0.8489	1.8818

**Table 9:** DOA estimation of nearby outer race by UCMA-ESPRIT for rolling element fault signal at 290 rpm.

Elevation Angle (°)	6.40	6.17	8.92	8.01	6.71
Azimuth Angle (°)	43.30 ^1^	−102.87	108.13 ^1^	−56.52	163.85 ^1^
Elevation Angle (°)	10.49	7.98	7.04	8.98	11.27
Azimuth Angle (°)	17.49	49.89	79.61	119.71	145.14

^1^ Phase ambiguity exists.

**Table 10:** DOA estimation of nearby inner race by UCMA-ESPRIT for rolling element fault signal at 290 rpm.

Elevation Angle (°)	4.70	4.22	4.42	7.45	6.35
Azimuth Angle (°)	−108.02	−56.66	−5.86	−134.11 ^1^	−71.14 ^1^
Elevation Angle (°)	4.62	6.34	6.39	5.56	6.33
Azimuth Angle (°)	151.99	−154.25	93.62 ^1^	−32.70	12.80

^1^ Phase ambiguity exists.

**Table 11:** DOA estimation of nearby outer race by CPCMA-ESPRIT for rolling element fault signal at 290 rpm.

Elevation Angle (°)	6.51	6.52	7.59	7.59	7.15
Azimuth Angle (°)	−142.16	−110.93	−80.06	−54.18	−20.03
Elevation Angle (°)	8.75	7.90	7.39	7.26	7.95
Azimuth Angle (°)	8.32	39.54	70.03	−83.00 ^1^	125.72

^1^ Phase ambiguity exists.

**Table 12:** DOA estimation of nearby inner race by CPCMA-ESPRIT for rolling element fault signal at 290 rpm.

Elevation Angle (°)	4.78	4.91	4.69	5.58	5.68
Azimuth Angle (°)	−113.39	−60.28	−5.51	50.04	105.66
Elevation Angle (°)	5.66	5.53	4.80	5.64	5.35
Azimuth Angle (°)	156.85	30.42 ^1^	−94.03	−39.87	14.83

^1^ Phase ambiguity exists.

**Table 13:** RMSE in ball pass location outer race (BPLO) by both arrays at 290 rpm.

UCMA		CPCMA	
Elevation Angle	Angle Interval	Elevation Angle	Angle Interval
1.6871	7.5369	0.7170	2.4965

**Table 14:** RMSE in ball pass location inner race (BPLI) by both arrays at 290 rpm.

UCMA		CPCMA	
Elevation Angle	Angle Interval	Elevation Angle	Angle Interval
1.0934	5.7557	0.3951	1.3909

**Table 15:** DOA estimation by UCMA-ESPRIT for outer race fault signal at 725 rpm.

Elevation Angle (°)	7.65	7.60	7.62	7.64	7.62	7.64	7.57
Azimuth Angle (°)	145.23	145.50	145.22	145.32	145.47	145.87	145.20

**Table 16:** DOA estimation by CPCMA-ESPRIT for outer race fault signal at 725 rpm.

Elevation Angle (°)	7.69	7.76	7.69	7.84	7.70	7.65	7.80
Azimuth Angle (°)	145.96	145.87	153.88	146.33	145.83	146.36	146.00

**Table 17:** Root-mean-square error (RMSE) in outer race defect located by both arrays at 725 rpm.

UCMA		CPCMA	
Elevation Angle	Azimuth Angle	Elevation Angle	Azimuth Angle
0.1719	4.6039	0.0855	3.9377

**Table 18:** DOA estimation by UCMA-ESPRIT for inner race fault signal at 725 rpm.

Elevation Angle (°)	4.55	4.52	5.72	6.05	4.81
Azimuth Angle (°)	−79.95	137.79 ^1^	−173.15 ^1^	54.30	98.68
Elevation Angle (°)	6.17	6.15	5.70	5.50	6.06
Azimuth Angle (°)	146.10	−163.41	−115.82	−68.55	−19.52

^1^ Phase ambiguity exists.

**Table 19:** DOA estimation by CPCMA-ESPRIT for inner race fault signal at 725 rpm.

Elevation Angle (°)	4.77	4.79	4.87	5.12	4.95
Azimuth Angle (°)	−89.59	−42.01	2.72	49.92	93.11
Elevation Angle (°)	5.64	4.95	5.57	5.28	5.55
Azimuth Angle (°)	−38.45 ^1^	9.74 ^1^	−121.43	−75.22	−27.71

^1^ Phase ambiguity exists.

**Table 20:** RMSE in inner race defect located by both arrays at 725 rpm.

UCMA		CPCMA	
Elevation Angle	Angle Interval	Elevation Angle	Angle Interval
0.6673	3.5591	0.3501	1.8104

## Data Availability

Not applicable.
